# Impact of Mated Female Nonproductive Days in Breeding Herd after Porcine Epidemic Diarrhea Virus Outbreak

**DOI:** 10.1371/journal.pone.0147316

**Published:** 2016-01-15

**Authors:** Jung-Da Lin, Chuen-Fu Lin, Wen-Bin Chung, Ming-Tang Chiou, Chao-Nan Lin

**Affiliations:** 1 Department of Business Administration, National Chung Cheng University, Chiayi, Taiwan; 2 Department of Veterinary Medicine, National Chiayi University, Chiayi, Taiwan; 3 Department of Veterinary Medicine, College of Veterinary Medicine, National Pingtung University of Science and Technology, Pingtung, Taiwan; 4 Animal Disease Diagnostic Center, College of Veterinary Medicine, National Pingtung University of Science and Technology, Pingtung, Taiwan; Cornell University, UNITED STATES

## Abstract

Porcine epidemic diarrhea virus (PEDV) is an important pathogen that has a significant economic impact on the swine industry by imposing a high rate of mortality in suckling piglets. However, limited information on the productivity values of gilts and sows infected with PEDV is available. Here, we evaluate the productivity index in gilts and sows during the 1-year period before (19 January 2013 to 18 January 2014) and after (19 January 2014 to 18 January 2015) a PEDV outbreak from a 2000-sow breeding herd in Taiwan. The farrowing rate (FR), return rate (RR), total pigs born per litter (TB), pigs born alive per litter (BA), weaning pigs per litter (WPL), pre-weaning mortality, percentage of sows mated by 7 days after weaning, weaning to first service interval (WFSI), mated female nonproductive days (NPDs), replacement rate of sows and sow culling rate were compared using productive records. The FR (-9.6%), RR (+9.8%), TB (-1.6), BA (-1.1), WPL (-1.1), sows mated by 7 days after weaning (-6.9%), WFSI (+0.8 days), NPDs (+6.9 days) and sow culling rate (+7.2%) were significantly different between the 1-year pre-PEDV outbreak period and the post-PEDV outbreak period. Impacts of the PEDV infection on the reproductive performance were more severe in pregnant gilts than in sows. In conclusion, these findings indicate that the outbreak of PEDV caused an increase in the rate of NPDs in breeding herds.

## Introduction

Porcine epidemic diarrhea (PED) is an important swine disease that causes a significant impact in most pig-producing countries [1]. The causative agent, the PED virus (PEDV), belongs to the genus *Alphacoronavirus*, family *Coronaviridae*, and order *Nidovirales* [1]. Although PEDV was first observed in Europe in the early 1970s [2], it has become an increasing problem worldwide, including in the Americas [3–5], Asia [6–11] and Europe [12, 13]. The devastating effect of PEDV infection is mainly due to the acute watery diarrhea and dehydration induced in infected pigs that not only leads to high (80–100%) mortality in neonatal piglets [3, 9] but also impairs the health and performance of the surviving pigs [14].

The impact of PEDV infection on the reproductive performance of gilts and sows depends on the period of pregnancy, during which females are exposed to the pathogen and the parity number [15]. The farrow rate (FR) (-3.8%), percentage of stillborn piglets per litter (+1.8%), and percentage of mummified fetuses per litter (+1.1%) were significantly different during the 4-month period of the PEDV outbreak compared with the same period in the year before the outbreak [15]. However, limited information on the productivity index of the gilts and sows that were exposed to the PEDV during the 1-year period of the PEDV outbreak is available. Mated female nonproductive days (NPDs) in the herd with the PEDV outbreaks have not been reported.

NPDs are the days that a mated sow or gilt is present in the herd and is neither gestating or lactating [16]. The formula for calculating NPD is NPD = 365−[(litter/female/year) × (gestation days + lactation days)]. Several factors affect the NPDs [16]: i) replacement gilt days, entry to first service, entry to culling and entry to death; ii) weaning-to-first service days (the number of days from weaning until a female is mated again); iii) first to repeat service interval (days to find re-cycling females after breeding); iv) weaning to removal period; and v) death loss and gestation days that do not result in farrowing. Therefore, NPDs represent key performance indicators of breeding herd performance.

The objectives of the present study were to investigate the effects between a 1-year period before and after PEDV outbreak on a sow’s reproductive traits on a commercial pig farm in Taiwan.

## Materials and Methods

### Ethics Statement

This was a retrospective study that did not require direct intervention, retrieval of clinical specimens or animal experiments. The pig owners provided written consent for data collection and publication. No specific permissions were required for the location of the data because the data were collected with swine management software.

### Study population

The present study was conducted on a 2000-sow commercial pig farm in the central region of Taiwan. The name of the pig farm is Mai-Chung Pig Farm with the following geographical coordinates (latitude/ longitude): 23°46′14.2″N and 120°14′56.0″E. On average, the productivity index of the study herd before the outbreak of PEDV was superior to that recorded in other breeding herds in Taiwan. The health of the herds was monitored by the herd veterinarian and the Animal Disease Diagnostic Center (ADDC), National Pingtung University of Science and Technology (NPUST). The majority of the females were crossbreed Landrace × Yorkshires that were produced from their own grandparent stock. The veterinarian recommended vaccinating gilts against foot-and-mouth disease virus (FMDV), classical swine fever virus (CSFV), Aujeszky’s disease (ADV), porcine parvovirus (PPV), porcine circovirus type 2 (PCV2) and atrophic rhinitis between 24 and 30 weeks of age in replacement gilts. Mass vaccinations for ADV, PCV2 and FMDV were conducted in the sows every 4 months, 6 months and year, respectively. Vaccinations of sows against CSFV and porcine reproductive and respiratory syndrome virus (PRRSV) were conducted on weaning day. No sows exhibited ADV, PCV2, PPV, PRRSV or bacterial abortion (*Streptococcus suis*, *Erysipelothrix* spp, *Leptospira interrogans*) in this breeding herd during the study period as monitored by the herd veterinarian and the ADDC, NPUST through molecular diagnosis and serological surveillance. The target replacement rate of the sows by gilts was approximately 48% annually. Sow culling due to age was planned to occur after the sixth parity, after the second return or vulva discharges at 14–21 days post-service. On 18 January 2014, PEDV infection was confirmed from this pig farm by ADDC, NPUST [9]. Farm immunization was performed using twice feedback with a 2-week interval for gilts and sows with approximately 10 ml of the homogenized intestines collected from PEDV-infected suckling piglets (1 piglet for 20 sows on average). After the first feedback, more than 90% of the gilts and sows showed clinical signs of anorexia, diarrhea and vomiting. Stool specimens were positive for PEDV using real-time PCR assays conducted by ADDC, NPUST. Less than 5% of the gilts and sows showed clinical signs of mild diarrhea and vomiting in the second feedback. Suckling piglet mortality reduced and PEDV-signs of gilts and sows stopped within 4 weeks after the first feedback immunization. Until early August 2014, pig owners worried about the reemergence of PEDV. Therefore, feedback of gilts and sows was conducted with approximately 10 ml of the homogenized frozen intestines from PEDV-infected suckling piglets (1 piglet for 100 sows on average) for three consecutive days. After this feedback event, more than 80% of the gilts and rarely sows showed clinical signs of mild anorexia, diarrhea and vomiting. No feedback immunization has been performed in this pig herd since August 2014. There were 20 and 8 sows (approximately 60% of them are primiparous sows) that were observed to have endemic PEDV infections in early December 2014 and September 2015, respectively. These PEDV infections were confirmed by ADDC, NPUST. During the post-PEDV period, all of the gilts and sows were exposed to PEDV by natural or/and feedback routes.

### Data

Data on gilt and sow reproductive traits were obtained from the swine management software of the herd from 19 January 2013 to 18 January 2015 (Porcitec 2009 version, AGRITEC). The collected data included sow identities, mating dates, mating results, number of days until the sows returned to estrus after mating, FR, return rates (RR), litters/mated female/year (LMFY), percentage of sows mated by 7 days after weaning, weaning to first service intervals (WFSI), farrowing intervals (FI), NPDs, replacement rate of sows, sow culling rate, total pigs born per litter (TB), pigs born alive per litter (BA), weaning pigs per litter (WPL) and pre-weaning mortality. The reproductive data from before and after the PEDV outbreak were collected during periods from 19 January 2013 to 18 January 2014 and from 19 January 2014 to 18 January 2015, respectively.

### Statistical analysis

NPDs of the different parities (1st, 2nd and >2nd) during the 1 year pre- (2013) and post- (2014) PEDV outbreak were compared using a two-way ANOVA for multiple comparisons. The average number of mated females, average parity of farrowed sows, number of matings, number of farrowings, FR, RR, number of abortions, LMFY, percentage of sows mated by 7 days after weaning, WFSI, FI, NPDs, replacement rates of sows and sow culling rates of pre- and post-PEDV outbreak periods were compared using a Mann-Whitney test. P values < 0.05 and <0.01 were considered to be statistically significant and highly significant, respectively.

## Results

### FR, RR and the number of abortions

**Fig 1:**
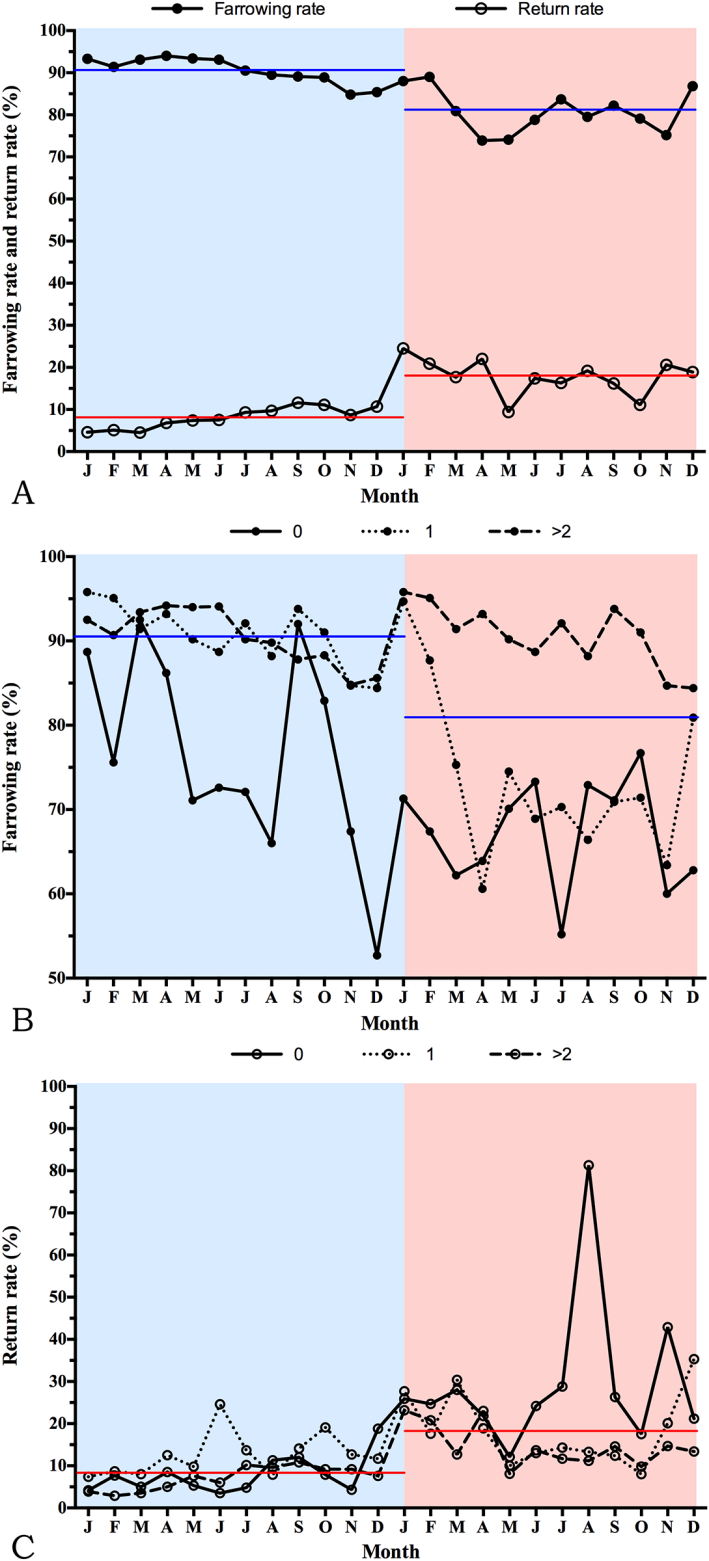
Comparison of farrowing rate and return rate between 1 year pre- (19 January 2013 to 18 January 2014) and post- (19 January 2014 to 18 January 2015) porcine epidemic diarrhea virus (PEDV) outbreak (A). (B) Farrowing rate of gilts (parity 0) and sow parities 1 and >2. (C) Return rate of gilts (parity 0) and sow parities 1 and >2. Blue and pink color represent pre- and post-PEDV outbreak, respectively. Blue and red lines represent the annual mean of farrowing rate and return rate, respectively.

**Fig 2:**
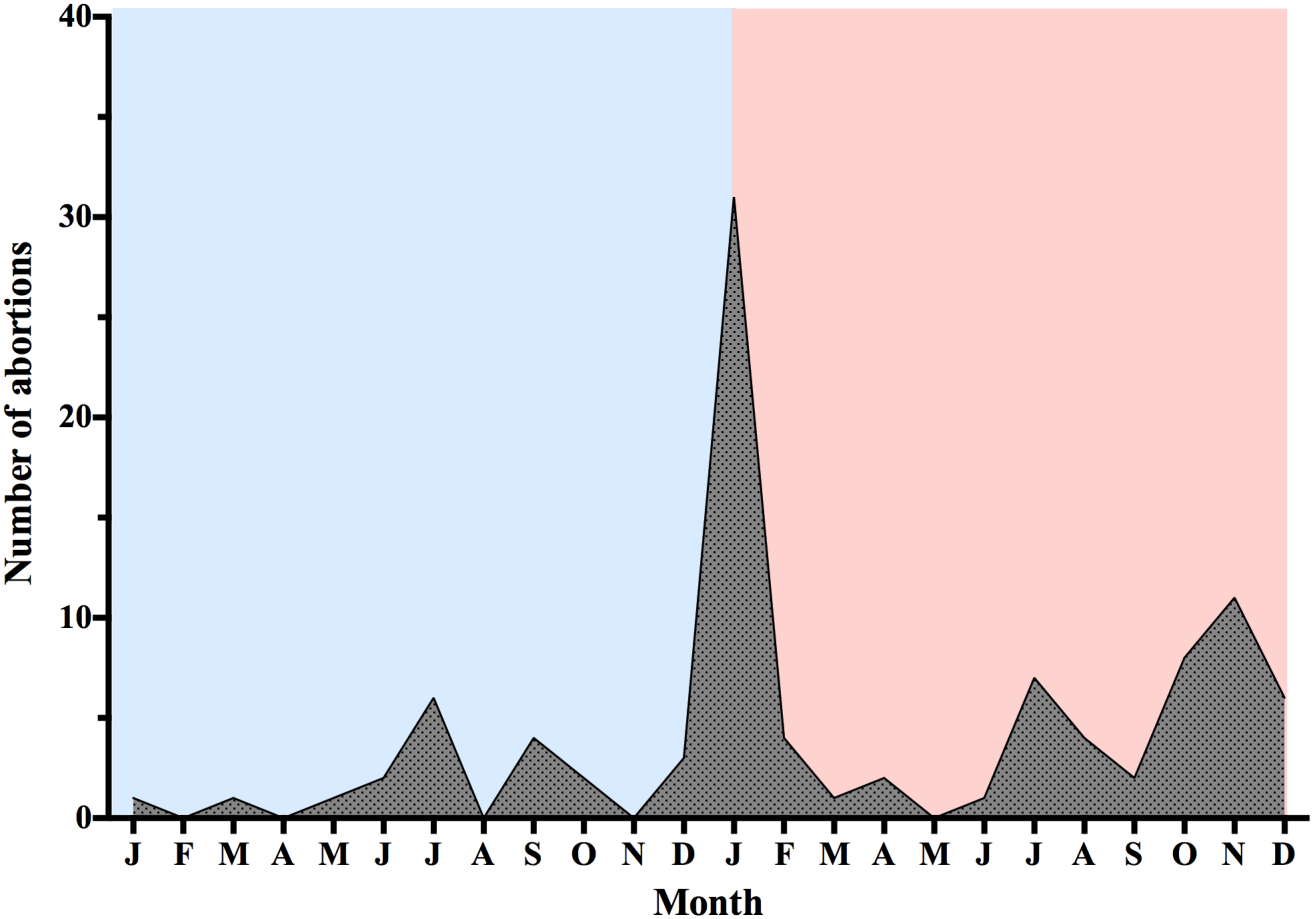
Comparison of the number of abortions between 1 year pre- (19 January 2013 to 18 January 2014) and post- (19 January 2014 to 18 January 2015) porcine epidemic diarrhea virus (PEDV) outbreak. Blue and pink color represent pre- and post-PEDV outbreak, respectively.

The productivity index of the sows in the herd during the 1-year period pre- and post-PEDV outbreak is presented in Table 1. The number of matings showed a 58- point increase during the post-PEDV outbreak period; however, the number of farrowings showed a 214-point decrease during this period. The average parity of the farrowed sows was significantly higher post-PEDV outbreak compared with the period before the PEDV outbreak (3.8 vs. 3.5). One year before the PEDV outbreak, the FR, RR and number of abortions in the herd were 90.5%, 8.1% and 20, respectively (Table 1). However, a 9.6 percentage point decrease in FR (P<0.001) (Table 1 and Fig 1A), 9.8 percentage point increase of RR (P<0.001) (Table 1 and Fig 1A) and a 4.8% increase of abortion rates (P = 0.0288) (Table 1) were observed after the PEDV outbreak (Table 1). Additionally, accounting for parity, the influence of the PEDV outbreak on the FR and RR was more pronounced in pigs having their initial pregnancies (Fig 1B and 1C). Interestingly, the number of abortions rapidly increased during the PEDV outbreak period (19 January to 18 February 2014) (Fig 2). The abortion rate (AR) after the PEDV outbreak was significantly higher than the AR before the PEDV infection (+4.8%, P = 0.028) (Table 1). Together, these results indicated that the reduction in reproductive performance was more severe in pregnant gilts than in pregnant sows during the post-PEDV outbreak period.

**Table 1:** Comparison of the productivity values between 1-year pre- (19 January 2013 to 18 January 2014) and post- (19 January 2014 to 18 January 2015) porcine epidemic diarrhea virus (PEDV) outbreak.

Production index	Pre-PEDV outbreak	Post-PEDV outbreak	Difference	P value^a^
Number of matings (average/month)	5873 (489.4)	5931 (494.3)	+ 58	0.809
Number of farrowings (average/month)	4786 (398.8)	4572 (381)	- 214	0.298
Average parity of farrowed sows	3.5	3.8	+ 0.3	0.012*
Farrowing rate (%)	90.5	80.9	- 9.6	<0.0001**
Return rate (%)	8.1	17.9	+ 9.8	<0.0001**
Percentage of multiple matings	99.7	98.7	- 1.0	0.643
Number of abortions (average/month %)	20 (1.7)	77 (6.5)	+ 57	0.028*
Litters/mated female/year	2.33	2.28	- 0.05	0.068
Sows mated within 7 days post-weaning (%)	84.2	77.3	- 6.9	0.012*
Weaning to first service interval (days)	5.4	6.2	+ 0.8	0.013*
Farrowing interval (days)	149.4	152.4	+ 3	0.003**
Nonproductive female days	42.4	49.3	+ 6.9	0.012*
Replacement rate of sows (%)	48.9	48.0	- 0.9	0.921
Sow culling rate (%)	32.2	39.4	+ 7.2	0.0575
Lactation length (days)	25.6	25.7	+ 0.1	0.751
Gilt pool (average/month)	1019 (84.9)	971 (80.9)	- 48	0.385

^a^ The Mann-Whitney test was used for comparisons between 1-year pre- and post-PEDV outbreak.

* and ** were considered statistically significant and very highly significant, respectively.

### Litter size at birth and weaning

TB, BA, total weaning pigs, WPL and pre-weaning mortality during the 1-year period pre- and post-PEDV outbreak are shown in Table 2. TB (P<0.001), BA (P<0.001) and WPL (P<0.001) decreased significantly after the PEDV outbreak compared with the 1-year period before PEDV infection. The suckling piglets infected with PEDV during the disease outbreak period (19 January to 18 February 2014) had a 41.7 percentage point increase in pre-weaning mortality (17.3% vs. 59%) compared with the same period in the year before the outbreak (19 January to 18 February 2013) (data not shown).

**Table 2:** Comparison of the total number of pigs born per litter, number of pigs born alive per litter and weaning pigs per litter between 1-year pre- (19 January 2013 to 18 January 2014) and post- (19 January 2014 to 18 January 2015) porcine epidemic diarrhea virus (PEDV) outbreak.

Production index	Pre-PEDV outbreak	Post-PEDV outbreak	Difference	P value^a^
Total pigs born/litter	13.7	12.1	-1.6	<0.0001**
Pigs born alive/litter	12.6	11.5	-1.1	<0.0001**
Total weaning pigs (average/month)	50,569 (4214.1)	43,371 (3614.3)	-7198	0.126
Weaning pigs/litter	10.7	9.6	-1.1	0.0003**

^a^ The Mann-Whitney test was used for comparisons between 1-year pre- and post-PEDV outbreak.

** was considered statistically very highly significant.

### NPDs, replacement rate of sows and culling rate

**Fig 3:**
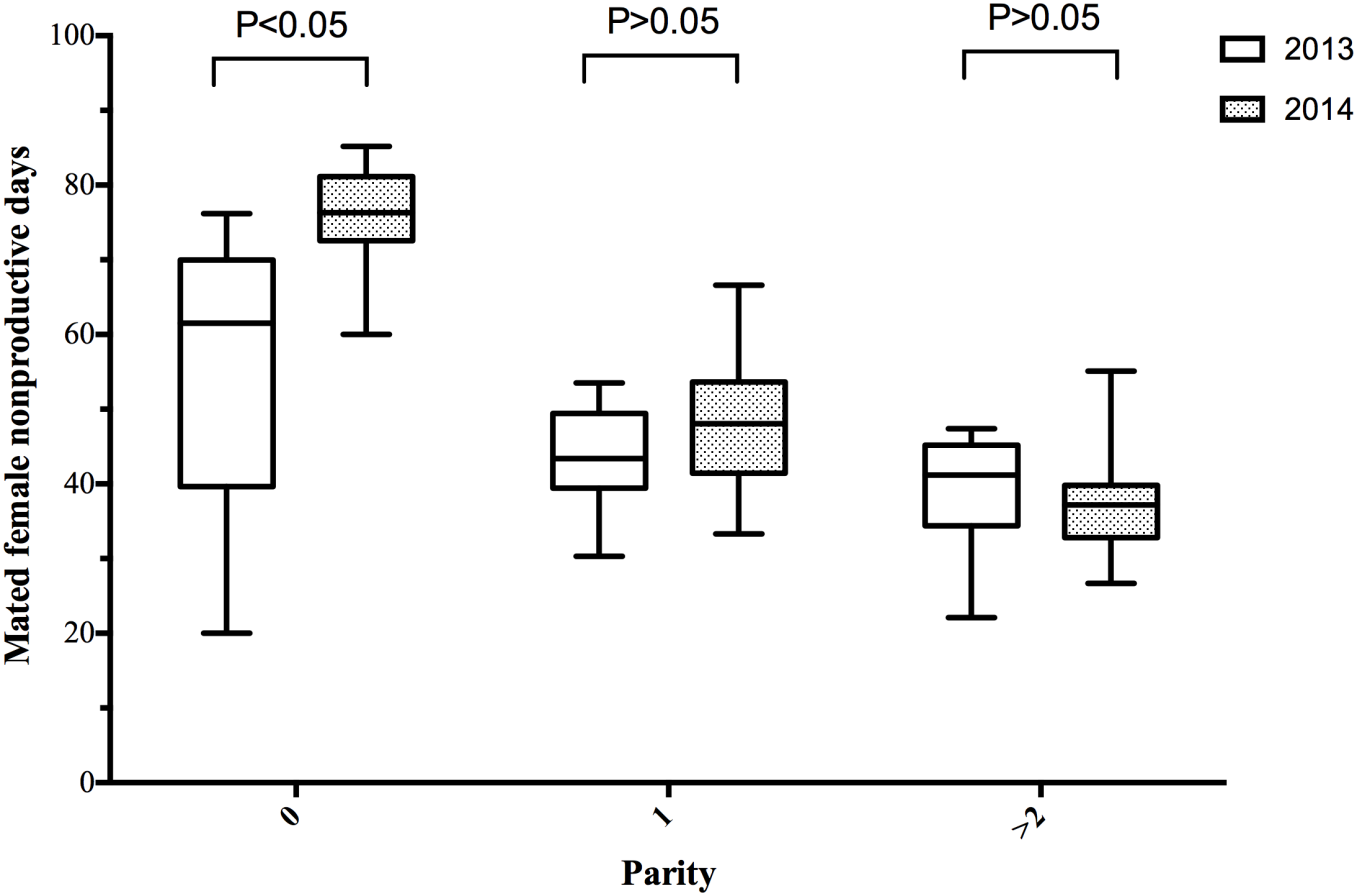
Comparison of mated female nonproductive days between 1 year pre- (19 January 2013 to 18 January 2014) and post- (19 January 2014 to 18 January 2015) porcine epidemic diarrhea virus (PEDV) outbreak.

**Fig 4:**
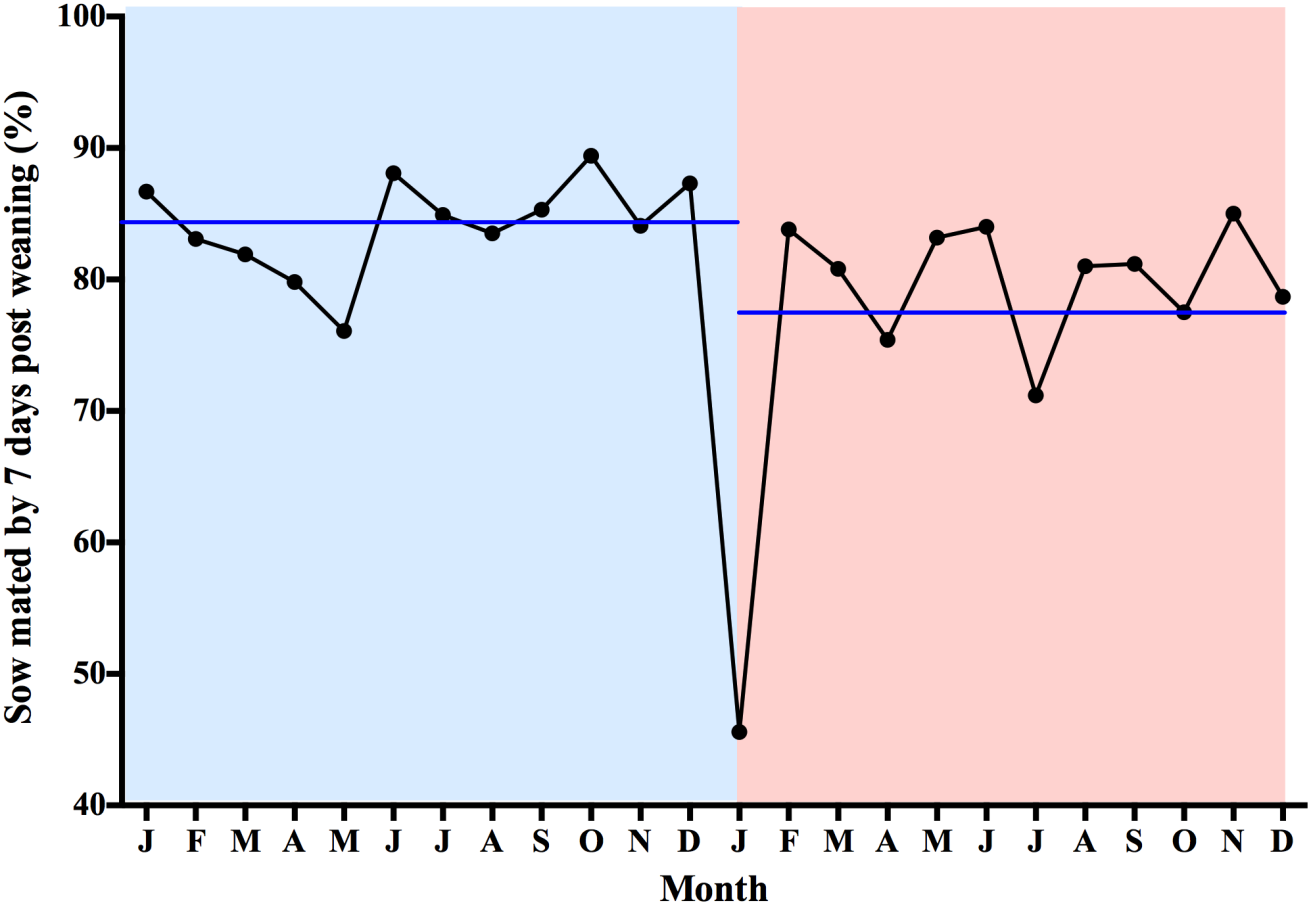
Comparison of the percentage of sows mated by 7 days post-weaning between 1 year pre- (19 January 2013 to 18 January 2014) and post- (19 January 2014 to 18 January 2015) porcine epidemic diarrhea virus (PEDV) outbreak. Blue and pink color represent pre- and post-PEDV outbreak, respectively. Blue line represents the annual mean of the percentage of sows mated by 7 days after weaning.

**Fig 5:**
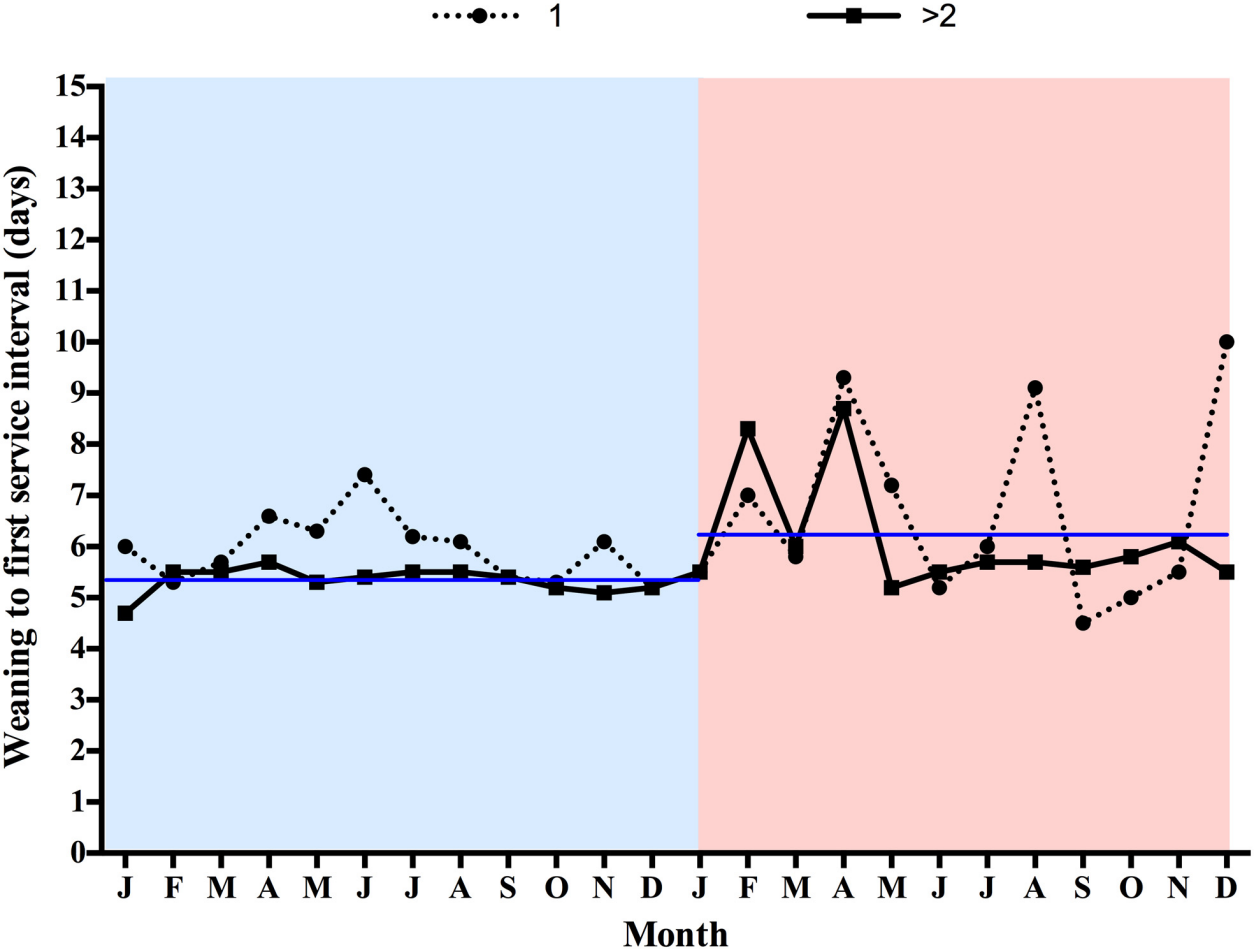
Comparison of weaning to first service interval in different sow parities (1 and >2) between 1 year pre- (19 January 2013 to 18 January 2014) and post- (19 January 2014 to 18 January 2015) porcine epidemic diarrhea virus (PEDV) outbreak. Blue and pink color represent pre- and post-PEDV outbreak, respectively. Blue line represents the annual mean of weaning to first service interval.

The percentage of sows mated within 7 days post-weaning, WFSI, FI, NPDs, LMFY, replacement rates of sows and sow culling rates are listed in Table 1. One year after the PEDV outbreak, we recorded a 6.9 percentage point decrease in the sows mated within 7 days after weaning (P = 0.0121) (Table 1), 0.8 percentage point increase in WFSI (P = 0.0131) (Table 1), 3-day increase in FI (P = 0.0035) and 6.9-day increase in NPDs (P = 0.0126) (Table 1), whereas the influence of the PEDV outbreak on the LMFY (P = 0.0681), replacement rate of sows (P = 0.9206) and sow culling rate (P = 0.0575) was not significantly different between the pre- and post-outbreak periods. In addition, when parity was taken into account, the influence of the PEDV outbreak on the NPDs was more pronounced in pigs with initial pregnancies (Fig 3). The percentage of sows mated within 7 days post-weaning was related to the WFSI, which is one of the factors that affected the NPDs. Interestingly, the percentage of sows mated within 7 days post-weaning declined (from 86.7% to 45.6%) during the PEDV outbreak period (19 January to 18 February 2014) compared with the same period in the year before the PEDV outbreak (19 January to 18 February 2013) (Fig 4). The WFSI was highly variable during the post-PEDV outbreak period compared with the period 1 year before the outbreak (Fig 5). In addition to this pig farm, we also analyzed the productivity index during the 1-year period before and after PEDV outbreaks from one Taiwanese farrow-to-finish herd (500 sows). The results showed that there were impacts on reproductive performance after PEDV infection. We recorded a 6.2-day increase in NPDs during the post-outbreak periods (data not shown). Taken together, these results indicate that there was a significant increase of the NPDs after the PEDV outbreaks occurred, especially in pregnant gilts.

## Discussion

The devastating effect of PEDV infection is primarily due to the acute watery yellowish diarrhea and dehydration, with mortality rates ranging from 80 to 100% in suckling piglets under 2 weeks of age [9]. However, only a few studies have attempted to assess the impact of PEDV infection on the reproductive and growth performance of sows [15] and surviving pigs [14], respectively. In the present study, we compared the productivity index of gilts and sows between 1 year pre- and post-PEDV outbreak in a Taiwanese breeding herd.

Comparison of the FR (80.9% vs. 87.5%), RR (17.9% vs. 5.0%), AR (6.5% vs. 2.7%), TB (-1.6 vs. +0.3), BA (-1.1 vs. -0.1) and pre-weaning mortality (59% vs. 49.2%) revealed that post-PEDV period effects were more severe than those observed in a study conducted in Thailand [15]. This finding may be due to the following: i) the different observation periods (a 1-year period in the present study vs. a 4-month period in the Thailand study); ii) the endemic PEDV outbreak was present in this herd after the pandemic outbreak of PEDV and iii) the different strains of PEDV (US-like strain in Taiwan vs. Chinese-like strain in Thailand) [9, 15, 17]. Overall, these two studies consistently observed that the influence of the PEDV outbreak on FR and RR was more pronounced in pigs that are early in their pregnancy.

NPDs are key performance indicators of breeding herd performance. Some factors that may affect NPDs include the following: i) replacement gilt timing, ii) weaning-to-first service days, iii) first service to repeat service intervals, iv) weaning to removal intervals, and v) death losses. To our knowledge, this is the first report showing the influence of PEDV on NPDs in gilts and sows. Factors that contributed to prolonging NPDs include increases in RR, number of abortions, percentage of sows mated within 7 days after weaning, WFSI and FI. In general, lactation levels declined during the PEDV outbreak, especially in infected herds with high suckling mortality. Incomplete uterine involution and tissue repair in early weaned sows contributed to the increased embryo loss in infected herds. The percentage of sows mated within 7 days after weaning rapidly declined (86.7% from 19 January to 18 February 2013 vs. 45.6% from19 January to 18 February 2014) during the PEDV outbreak period, resulting in an increase in WFSI. A significant increase in AR was also observed by Pijpers et al. [18] and Olanratmanee et al [15], although the mechanism underlying RR and FR is not known. Previously, studies have shown that lactation intervals not only affect the average number of days from weaning to estrus but also the pregnancy rates and number of live embryos per female [19]. This fact explains why the FR and TB significantly decreased after the PEDV outbreak in the present study.

Our results revealed that the influence of the PEDV outbreak on NPDs was more pronounced in primiparous sows. The primiparous sows exhibited severe clinical signs of anorexia, diarrhea and vomiting when infected with PEDV, while young sows were still utilizing nutrients for both growth and maintenance of the reproductive function. Previous studies have reported that i) increasing feed intake during lactation can increase luteinizing hormone secretion and reduce the weaning-to-estrous and farrowing-to-estrous intervals in primiparous sows [20], ii) protein (lysine) restriction throughout lactation alters circulating concentrations of somatotropic hormones and insulin at the end of lactation and has a negative impact on the post-weaning ovulation rate in primiparous sows [21], iii) low lysine levels in primiparous lactating sows impaired follicular development and reduced the ability of follicles to support oocyte maturation [22] and iv) low-parity sows were more sensitive to lactational feed intake than high-parity sows in terms of WFSI [23]. We recorded a 0.8-day increase in WFSI post PEDV infection (Table 1), occurring prominently in primiparous sows (Fig 5). These facts may explain why the influence of the PEDV outbreak on NPDs was more pronounced in primiparous sows. Additionally, maximizing feed intake during lactation is critical to improve the overall sow reproductive performance including productivity and longevity [24]. Until now, PEDV has not been considered as direct cause of reproductive problems. Therefore, inadequate feed and nutrient intake when PEDV infection may be cause the excessive body weight loss that can lead to short-term reproductive problems such as extended WFSI and smaller subsequent litter size. In the long run, problems such as higher culling rate of the breeding herd will result in low average parity; reduced pigs weaned per reproductive lifetime and increased production cost. In general, all phases of the reproductive cycle are related [24]. Overall, these facts may provide an explanation to why PEDV infection has such long-term impact on sow reproductive performance.

The impact of PEDV on NPDs in gilts and sows may be controlled or improved by the following methods: i) reducing the abortion rate (performance of the feedback immunization must be avoided when the gilts and sows are in their first month of pregnancy [15]), ii) decreasing lactation length [25], iii) decreasing the percentage of gilts in the breeding herd inventory [25], iv) decreasing the female culling rate [25–27] and v) increasing the percentage of multiple matings [25–27]. The pig owners did not alter the gilt and sow management, including lactation length (25.6 days vs. 25.7 days)(Table 1), percentage of multiple matings (99.7% vs. 98.7%)(Table 1), parity of culled sows (Sow culling due to age was planned to occur after the sixth parity), and percentage gilts in the breeding-female inventory (gilt pool: 1019 vs. 971), etc. (Table 1). Therefore, the reproduction indices were normal until the second year after the first PEDV outbreak. We recorded a 91.7 percentage of sows mated within 7 days post-weaning, 12.9 percentage of RR, 5.8-day of WFSI and 40.5-day of NPDs during the second year of the PEDV outbreak (19 January 2015 to 18 November 2015) (S1 Table). Overall, the control and improvement of impact of PEDV on NPDs in gilts and sows may alter the management of females.

## Conclusions

Outbreaks of PEDV not only lead to high mortality in neonatal piglets and a poorer performance of surviving pigs but also impair the productivity index in gilts and sows. This is the first report to show the influence of PEDV on NPDs in gilts and sows. These findings should contribute to an understanding of the effects of PEDV outbreak post-infection on sow herds and to an identification of ways to curtail losses as a result of this disease.

## Supporting Information

S1 Table Comparison of the productivity values between 1-year pre- (19 January 2013 to 18 January 2014), first year post- (19 January 2014 to 18 January 2015) and the second year post- (19 January 2015 to 18 November 2015) porcine epidemic diarrhea virus (PEDV) outbreak. (DOCX)
