# Supplementary material for: Impact of Mated Female Nonproductive Days in Breeding Herd after Porcine Epidemic Diarrhea Virus Outbreak
Source: PLoS One. 2016 Jan 15;11(1):e0147316. doi: 10.1371/journal.pone.0147316 (PMC4714882; doi:10.1371/journal.pone.0147316)
Supplement: S1 Table — (DOCX) [file pone.0147316.s001.docx]

**S1 Table. Comparison of the productivity values between 1-year pre- (19 January 2013 to 18 January 2014), first year post- (19 January 2014 to 18 January 2015) and the second year post- (19 January 2015 to 18 November 2015) porcine epidemic diarrhea virus (PEDV) outbreak.**

| Production index | Pre-PEDV outbreak | Post-PEDV outbreak | |
| --- | --- | --- | --- |
|  |  | First year | Second year |
| Return rate (%) | 8.1 | 17.9 | 12.9 |
| Litters/mated female/year | 2.33 | 2.28 | 2.30 |
| Sows mated within 7 days post-weaning (%) | 84.2 | 77.3 | 91.7 |
| Weaning to first service interval (days) | 5.4 | 6.2 | 5.8 |
| Nonproductive female days | 42.4 | 49.3 | 40.5 |
| Replacement rate of sows (%) | 48.9 | 48.0 | 49.0 |
